# The Origin and Evolution of the Archaeal Domain

**DOI:** 10.1155/2014/915828

**Published:** 2014-06-04

**Authors:** Gustavo Caetano-Anollés, Kyung Mo Kim

**Affiliations:** ^1^Evolutionary Bioinformatics Laboratory, University of Illinois, Urbana, IL 61801, USA; ^2^Microbial Resource Center, Korea Research Institute of Bioscience and Biotechnology, Daejeon 305-806, Republic of Korea

With this special issue on the origin and evolution of Archaea we honor and celebrate the life and impactful contributions of Carl Woese (July 15, 1928-December 30, 2012). Carl was born and raised in Syracuse, New York. His undergraduate studies were in Amherst College and his graduate training in Yale. Sol Spiegelman brought him to the University of Illinois at Urbana-Champaign, where he unfolded a brilliant career. Carl was inspired by the originality of his mentor, Ernest C. Pollard, the tradition of biological form of D'arcy Thompson, the charisma of Francis Crick, the evolutionary tempo-mode perspective of G. G. Simpson, and the foresight of Darwin and Wallace. He understood the centrality of evolution in our understanding of biology and championed this perspective as he explored the molecular makeup of the translation machinery. His insightful mind is responsible for the discovery of the archaeal domain and for transforming comparative views of microbial diversity into an overarching evolutionary framework. Archaea constitutes the third domain of life, a remarkable group of akaryotic microbes with unique biochemical and genomic features, some of which resemble those of eukaryotes. Their habitats and lifestyles are very diverse, from extremophiles living in harsh environments to soil and marine mesophiles, from free-living microbes to gut-inhabiting methanogens and symbionts.

Carl's work did not only result in the definition of a new “urkingdom,” originally named by him as “archaebacteria,” but his insights prompted an appreciation (and respect) for the incredible microbial diversity of the biosphere. He battled the establishment to make way to a redefined microbiological science that treasured evolutionary thinking and acknowledged the centrality of microbes in the global ecosystems of our planet. He was also a harsh critic of the field of biology in general. He felt our biological views are still governed by reductionistic biases inherited from the genetic and genomic revolutions of last century, which could not identify any important questions left to answer. Furthermore, he strongly felt the biological sciences were devoted and defined by the application side, that is, by focusing on providing “service to society” through bioengineering instead of acting as “society's teacher” of man's place in the universe. A number of unsolved problems that are central to understanding life remain to be answered, and Carl posed some of the basic questions from the very beginning. What were life's origins? How did molecular and organismal complexity unfold? What are the ultimate governing principles of life? He recognized the limitations of the primacy of a genetic, molecular biology and mechanistic outlook that was gene-centered and prompted an exploration of biological complexity and emergence of biological organization within an evolutionary and physics framework. He recognized the importance of the proteinaceous backbone of life and how its design and function is delimited by the genetic code, translation, and its complex regulatory control.

In this special issue we bring back some of Carl's basic unanswered questions. While it is becoming clear that the archaeal domain may have an independent evolutionary history, its origin and links to the other two domains of cellular complexity remain contentious, as well as its placement in the tree of life. The question demands urgent attention. Several contributions of this special issue tackle important aspects of the origin, diversity, and evolution of the archaeal domain.

A review article by A. Spang et al. comprehensively describes current hypotheses on the relationships of the three domains and evaluates archaeal diversity and evolution using recent genomic data (e.g., metagenomes and single-cell genomes). P. Forterre also evaluates the contemporary scenarios for the origins of the three domains. Archaeal ancestor scenarios and the fusion hypothesis are criticized. Interestingly, he brings the evolutionary role of the virosphere to explain the diversification of the three domains from the last universal common ancestor of life. A. Nasir and G. Caetano-Anollés explore a novel comparative genomic framework that makes the vertical horizontal evolutionary contributions explicit, and G. Caetano-Anollés et al. advance structural phylogenomic analyses of protein and nucleic acid structures and their associated functions. These approaches reveal that Archaea is the most ancient domain, which prompts a careful reevaluation of current phylogenetic methodologies and our understanding of the rooting of the tree of life.

D. S. Shin et al. review the robustness of archaeal proteins against extremophilic environments at the protein 3-dimensional structural level. C. J. Reed et al. describe how archaeal species can be adapted into thermophilic, psychrophilic, piezophilic, and halophilic environments by characterizing the biophysical property of archaeal proteins. Both studies emphasize the importance of archaeal structural biology for understanding human biology with medical and industrial impacts.

G. Borrel et al. present a bioinformatics analysis of three genomes from a newly identified order of methanogens and find the pyrrolysine (22nd amino acid) coding system. The phylogenetic analysis indicates that this genomic feature is conserved in both archaeal methanogens and bacteria, which can be an example of continuing evolution of the genetic code directed by metabolic requirements. On another front, L. S. Yafremava et al. study amino acid substitution patterns in the protein domains of nonbarophilic and barophilic* Pyrococcus* species and reveal that barophily is a very ancient trait that unfolded with the early evolution of the genetic code during early adaptation to deep ocean environments.

J. R. Peterson et al. use many different state-of-the-art approaches (e.g., SiMPull and RNA-Seq) to quantitatively characterize the methanogenesis pathways and translational machinery of the methanogen* Methanosarcina acetivorans*. This bioinformatics modeling can be a first step to establish new archaeal model systems, very much as* E. coli* is used for bacteria.

Taken together, articles highlight patterns and processes responsible for archaeal diversity at genetic, genomic, biochemical, physiological, and ecological levels. It is our intention that the work presented here will stimulate further evolutionary thinking, following Carl's pioneering and unorthodox spirit.

*Gustavo Caetano-Anollés* *Kyung Mo Kim*

